# The Effect of Dietary Mushroom *Agaricus bisporus* on Intestinal Microbiota Composition and Host Immunological Function

**DOI:** 10.3390/nu10111721

**Published:** 2018-11-09

**Authors:** Gloria I. Solano-Aguilar, Saebyeol Jang, Sukla Lakshman, Richi Gupta, Ethiopia Beshah, Masoumeh Sikaroodi, Bryan Vinyard, Aleksey Molokin, Patrick M. Gillevet, Joseph F. Urban

**Affiliations:** 1 Diet Genomics and Immunology Laboratory, Beltsville Human Nutrition Research Center, Agricultural Research Service, U.S. Department of Agriculture Northeast Area, Beltsville, MD 20705, USA; newbyeol@gmail.com (S.J.); Sukla.Lakshman@ars.usda.gov (S.L.); Ethiopia.Beshah@ars.usda.gov (E.B.); Aleksey.Molokin@ars.usda.gov (A.M.); Joe.Urban@ars.usda.gov (J.F.U.J.); 2 Microbiome Analysis Center, George Mason University, Science & Technology Campus, Manassas, VA 20108, USA; rgupta11@masonlive.gmu.edu (R.G.); msikaroo@gmu.edu (M.S.); pgilleve@gmu.edu (P.M.G.); 3 Statistics Group, Agricultural Research Service, U.S. Department of Agriculture Northeast Area, Beltsville, MD 20705, USA; Bryan.Vinyard@ars.usda.gov

**Keywords:** mushroom, microbiome, immune response, macrophage, 16S rDNA

## Abstract

A study was designed to determine the potential prebiotic effect of dietary mushrooms on the host immune response, and intestinal microbiota composition and function. Thirty-one six-week-old pigs were fed a pig grower diet alone or supplemented with either three or six servings of freeze-dried white button (WB)-mushrooms for six weeks. Host immune response was evaluated in peripheral blood mononuclear cells (PBMC), and alveolar macrophages (AM) after stimulation with *Salmonella typhymurium*-Lipopolysaccharide (LPS). Isolated DNA from fecal and proximal colon contents were used for 16S rDNA taxonomic analysis and linear discriminant analysis effect size (LEfSe) to determine bacterial abundance and metabolic function. Pigs gained weight with no difference in body composition or intestinal permeability. Feeding mushrooms reduced LPS-induced *IL-1β* gene expression in AM (*P* < 0.05) with no change in LPS-stimulated PBMC or the intestinal mucosa transcriptome. LEfSe indicated increases in *Lachnospiraceae*, *Ruminococcaceae* within the order Clostridiales with a shift in bacterial carbohydrate metabolism and biosynthesis of secondary metabolites in the mushroom-fed pigs. These results suggested that feeding WB mushrooms significantly reduced the LPS-induced inflammatory response in AM and positively modulated the host microbiota metabolism by increasing the abundance of Clostridiales taxa that are associated with improved intestinal health.

## 1. Introduction

Mushrooms contain many bioactive components including polysaccharides, glycoproteins, proteins, lipids, and secondary metabolites [1]. Polysaccharides composed of glucose, mannose, galactose, fucose, arabinose, glucuronic acid, and β-d-glucan are the most potent substances of mushrooms that show demonstrable beneficial properties such as antioxidant [2], immune-stimulatory [3,4] lipid lowering [5,6], and anti-tumor activity [7,8,9]. However, when discussing the biological activities of whole mushroom or its extracts, chemical structure, and extraction protocols need to be considered as water extracts usually activate immune cells whereas ethanol extracts inhibit them [10]. Thus, processing of the mushroom may determine the effect produced on immune cells, possibly due to different solubility and potency of specific compounds [10,11]. Edible mushrooms have been studied extensively for their immune modulating properties in animal models including β-glucan-induced anti-inflammatory effects [3,12], enhanced NK cell activity [11,13], improved dendritic cell (DC) maturation and function [14], increased cytokine production [15], increased protective immunity from Salmonella vaccination [16], and anti-inflammatory effects in patients with ulcerative colitis and Crohn’s disease [17,18,19,20].

Health promoting prebiotic effect has been shown after feeding some medicinal [21,22] and white button (WB)-raw mushrooms [23,24,25] in animal models.

The gastrointestinal (GI) microbiota perform numerous biological activities for the host [26] including facilitating nutrient availability, energy harvest and carbohydrate metabolism, and processing indigestible plant components [27]. Metabolic enzymes encoded in microbial genomes and released in the intestine will degrade dietary polysaccharides into short chain fatty acids (SCFA) that induce systemic anti-inflammatory activities on the host [25,28] and synthesize essential amino acids and vitamins [29].

Pigs have been extensively used as a model species to study human physiology and responses to disease. The similarities in size, anatomy, physiology, and metabolism of the digestive system of swine and humans, especially related to the dynamic shifts in the intestinal metagenome [30], nutrient digestibility [31,32], and intestinal epithelial cell renewal [33], are supportive of longitudinal studies to characterize the effect of feeding WB-mushrooms on intestinal microbiota structure, metabolic function, and host immune responses. Pigs are omnivorous, and their early developmental period is responsive to nutrient and metabolic modulation due to rapid growth like in humans [34,35]. Metagenome sequencing data derived from pig fecal DNA overlap with human functional pathways supporting the use of pigs for biomedical research of the host microbiome [36]. Therefore, the purpose of this study was to evaluate the effect of feeding WB mushrooms, the most commonly consumed edible mushroom in the United States [37], on the host gut microbiome composition and function and an immune response to bacterial Lipopolysaccharide LPS when consumed at two different doses for six weeks.

## 2. Experimental Design

### 2.1. Animals and Diets

All animal experiments and procedures were conducted in accordance with guidelines established and approved by Beltsville Area Animal Care and Use Committee under protocol 13-028. Fresh raw white button (WB) mushrooms were generously provided by To-Jo Mushrooms (To-Jo Mushrooms, Inc., Avondale, PA, USA). Immediately after arrival, fresh mushrooms were freeze dried for 10 days and ground at a grain mill before incorporation into diets. The nutrient component of mushroom was analyzed by Eurofins (Eurofins Scientific Inc., Des Moines, IA, USA) (Table 1). Thirty-one six-week-old pigs were obtained from the Oak Hill Genetics Farms, Ewing, IL, USA. Pigs were randomized by weight into three experimental treatment groups (*n* = 10–11/group). Group I was fed a regular pig grower diet. Group II was fed a regular pig grower diet supplemented with three servings of freeze-dried WB mushrooms equivalent to 75 g of fresh WB mushrooms fed to a human weighing 65–70 kg [38], and Group III was fed an equivalent of six servings equivalent to 150 g of fresh WB mushrooms (Table S1). Pigs were maintained on these diets for six weeks. Body weights were measured at zero, three, and six weeks of the study. Total body composition and bone mineral density in the femur was determined by a dual-energy X-ray absorptiometry (DXA) scan (Lunar Prodigy, GE HealthCare, Chicago, IL, USA) two days before the end of the intervention [39].

### 2.2. Immune Parameters

Peripheral blood mononuclear cells (PBMC) were isolated from whole blood samples collected in Ethylene-Diamine-Tetra-Acetic acid (EDTA)-anti-coagulant tubes after six-week of the diet study and were cultured with *Salmonella typhymurium*-Lipopolysaccharide (LPS) (10 ng/mL) (Sigma, St Louis, MO, USA) to determine ex-vivo cytokine production. Pigs were euthanized at the end of study and alveolar macrophages (AM) were isolated from lungs for phagocytosis assay [40] and for measuring LPS-induced cytokines released into culture supernatants and the gene expression of the cultured-cells [41,42]. Cytokine production was quantified using a porcine cytokine/chemokine magnetic bead panel for detection of GM-CSF, IFNγ, IL1α, IL1β, IL1RA, IL-2, IL-4, IL-6, IL-8, IL-10, IL-12, IL-18, and TNFα (Milliplex Map Kit, EMD Millipore Corporation, Billerica, MA, USA) on the Luminex-200 system (Luminex Corporation, Austin, TX, USA). Cytokine/Chemokine analyte concentration was calculated after adjusting median fluorescent intensity values to a standard curve per each analyte. The phenotype of PBMC and isolated Peyer’s patch lymphocytes was determined by flowcytometry (BD-FACS, Becton Dickinson, San Jose, CA, USA) [43].

### 2.3. Ex-Vivo Measurement of Intestinal Epithelial Cell Function

Three segments of jejunum mucosa (0.126 cm^2^) were stripped of smooth muscle and mounted on polycarbonate filters (diameter, 12 mm; pore size, 0.4 μm) in trans-well plates (Costar 3460, Corning Inc., Corning, NY, USA) containing Dulbecco’s Modified Eagle’s Medium DMEM-F12 with 10% fetal calf serum and incubated at 37 °C with 5% CO_2_ in air. The basolateral and apical compartments were filled with 1.5 and 0.5 mL of culture medium, respectively. *Trans*-epithelial electrical resistance (TEER) was measured in triplicate [44] using an ohm/voltmeter (EVOM, World Precision Instruments, Sarasota, FL, USA) in a six chamber-micro-snap-well system exposing the luminal side of the tissue. Following a 5-min equilibration, TEER was regularly registered every 30 min for 90 min (0, 30, 60, and 90 min) of treatment. Resistance values were calculated in Ω·cm^2^ by multiplying the resistance values by the filter surface area. Results are expressed as the percentage of the basal (control) value.

### 2.4. Intestinal Sample Processing

Additional segments from jejunum (1 mm^3^), next to the segment collected for TEER assay (mid-section of small intestine), and proximal colon mucosa were collected at necropsy and immediately frozen in liquid nitrogen and kept at −80 °C. RNA was extracted and cDNA processed for expression analysis of genes associated with intestinal mucosal integrity (tight junctions, occludins, and claudins) and intestinal transporter activity (glucose transporters) in jejunum as previously described [45]. Proximal colon mucosa derived RNA was also processed and sequenced as previously described [42].

### 2.5. Fecal Specimen Collection and Processing for 16S rDNA Amplicon Multi-Tag Sequencing

Fresh fecal samples were directly collected from the rectum of pigs using a cotton swab to stimulate defecation. A 5 g aliquot of each fecal sample was collected in a sterile 50 mL plastic tube before starting the dietary intervention at day 0 (baseline) and six weeks later. Proximal colon contents were also collected at necropsy at the end of the intervention. Samples were stored at −80 °C until further processing for DNA extraction [46]. A 10 ng-aliquot of extracted DNA was used for sequencing the first two variable regions of the 16S rDNA using Ion Torrent technology [47,48]. Ribosomal Database Project (RDP)11 analysis was used to identify the taxa present in each sample by classifying the sequences against version 11 of the RDP. Fifth level of taxa assignment (genus) was used to further identify differences in treatment groups. The operational taxonomic unit (OTU)s were also defined using Quantitative Insights into Microbial Ecology (QIIME 2) pipeline [49] and used in the calculation of diversity to predict metabolic function. Overall differences among dietary intervention groups were evaluated by principal component analysis (PCoA) using the Qiime pipeline [50] which relates the distribution of microbes among larger communities to better understand the biological factors that may drive these relationships. The distance matrix is constructed using UniFrac values that measure phylogenetic distances between sets of data that are represented in a two-dimensional space to facilitate visualization of differences among groups [51].

### 2.6. Predicted Metabolic Profiles of Microbiome

OTUs were normalized for number of 16S rDNA gene copies and used to predict metagenome functional content using the Phylogenetic Investigation of Communities by Reconstruction of Unobserved States (PICRUSt) [52]. PICRUSt uses existing annotations of genes based on bacterial evolutionary information as well as 16S gene copy numbers from Integrated Microbial Genomes database to predict metagenomes from 16S rDNA data [53]. This methodology predicts metabolic function of the microbiome with an average correlation of approximately 0.8 between inferred and measured gene content [52].

### 2.7. Statistical Analyses

Shannon diversity index was used as a measure of microbiota diversity within each treatment group. The index reflects the number of different species present (richness) and the relative abundance of each species (evenness) in the fecal samples. The mean and standard error (SE) and significance were calculated using a non-parametric Wilcoxon ranked test. The linear discriminant analysis effect size (LEfSe), an algorithm for biomarker discovery that identifies enrichment of abundant taxa or function between two or more groups, was used to compare all taxa at different taxonomic levels simultaneously (i.e., phylum, class, order, family, and genus) between treatment groups. The non-parametric Kruskal–Wallis statistical test was used to compute differences among treatment groups and then paired Wilcoxon rank sum tests among subgroups. This method uses a linear discriminant analysis (LDA) model with continuous independent variables to predict one dependent variable and provides an effect size for the significantly different taxa or metabolic function based on relative differences between variability and discriminatory power [54]. Unless stated otherwise, alpha values of 0.05 were used for the Kruskal–Wallis rank sum test, and a threshold of >2.0 was chosen for logarithmic LDA score display. A series of bar graphs were constructed to show the relationship between significantly different metabolic functions or taxa at different phylogenetic levels differentiating clades with a common ancestor [54]. Cytokines produced by LPS-stimulated PBMC and AM were analyzed based on a normal distribution model with a Sidak false discovery protection method. A treatment × time-ANOVA model was used for analysis of TEER data. Significance among levels of time for each treatment or for significance among treatments at each time were calculated. Means comparisons used a comparison-wise error rate, α_cer_ = 0.0102 (10 pairwise comparisons among the 5 times). Significant differences are reported with *p* < 0.05.

Determination of differentially expressed genes (DEG) were calculated using Bioconductor packages: edgeR [55] based on negative binomial generalized linear model. Gene counts derived from each dietary treatment group were compared among stimulant treatments and used to determine which common genes were differentially expressed. *P* values were corrected using the Benjamini–Hochberg false discovery rate adjustment [56]. A difference in gene expression was considered significant if the adjusted FDR *p*-value was < 0.1. Biological network analysis was performed using Ingenuity Pathway Analysis (IPA) (v 9.0 Ingenuity Systems, Mountain View, CA, USA) to predict potential biological processes, pathways, and molecules affected by DEGs. Networks of these focus genes are algorithmically generated based on their connectivity and number of focus genes. The more focus genes involved, the more likely the association is not due to random chance. To identify the networks that are highly expressed, IPA computes a score per the fit of the genes in the data set. This score is generated using a *p*-value calculation determined by a right-tailed Fisher’s exact test, and it is displayed as the negative log of that *p*-value. This score indicates the likelihood that the fit of the focus genes in the network could be explained by chance alone. A score of 2 indicates that there is a 10^−2^ chance that the focus genes are grouped together in a network by chance. *Z*-score serves as both a significance measure and a predictor of the activation state of the gene: activated (*Z* value > 2) or inhibited (*Z* value < 2) [57]. The goal was to identify those biological processes and functions that were likely to be casually affected by up- and downregulated genes.

## 3. Results

### 3.1. Clinical Signs

All pigs gained weight over the six weeks of the study with no differences in carcass fat deposition or bone mineral density composition in right femur between the three groups (Figure S1). There were no differences in distribution of the phenotype of Peyer’s patch cells including CD4 (T helper cells), CD8 (T cytotoxic cells), CD3 (T cells), CD25 (activation antigen), SLADQ (antigen presentation/activation), CD21 (B-cells), CD203 (macrophages), and CD14 (monocytes) (Figure S2A) or PBMC (data not shown) using flow cytometry. No significant differences were detected in relative phagocytic activity of AM from the different treatment groups (Figure S2B).

### 3.2. Immune Response Evaluation

**Figure 1:**
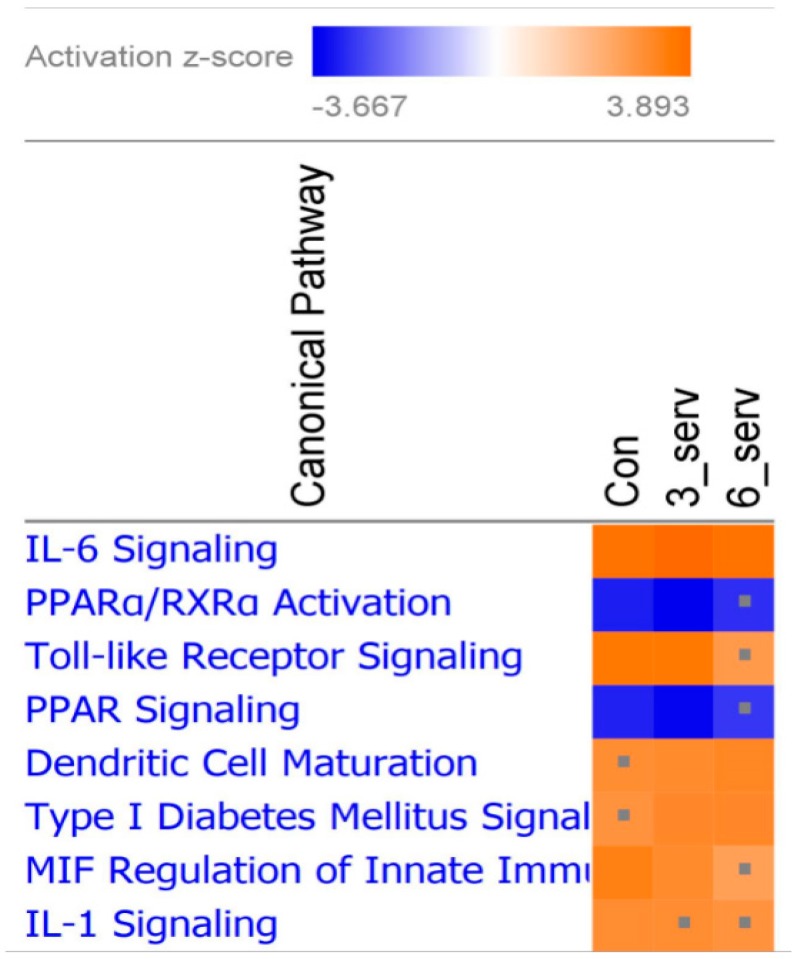
Canonical pathways constructed from differentially expressed genes induced in alveolar macrophages after Lipopolysaccharide (LPS) stimulation. Heatmap displays the top significant canonical pathways affected by dietary interventions. The orange and blue colored squares indicated predicted pathway activation or predicted inhibition, respectively, using a *z*-score threshold of 3.0. The significance indicates the probability of association of molecules from the dataset with the canonical pathway by random chance. Represents pathways with *z*-score less than 3.0.

**Figure 2:**
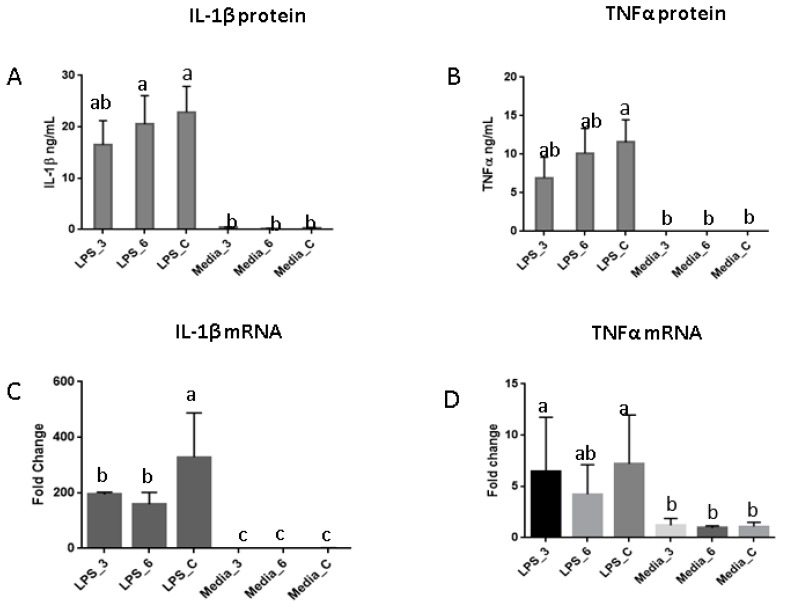
LPS-induced Interleukin 1 beta (IL-1β) and Tumor necrosis factor alpha (TNFα) in cultured alveolar macrophages (AM). (**A**,**B**) Bar plots representing IL-1β and TNFα protein produced by AM derived from pigs fed three servings, six servings or non-supplemented control diets and cultured with 5% porcine serum-complete media with *Salmonella typhimurium* LPS (10 ng/mL) (three bars on the left) or complete media without LPS (three bars on the right). (**C**,**D**) Gene expression for IL-1β and TNFα mRNA in AM measured by Real time-Polymerase Chain Reaction (RT-PCR). Bars represent a fold change of mean difference among dietary groups. Means with different superscripts are significantly different (*p* < 0.05).

RNA sequencing analysis identified 1290 common genes that were differentially expressed (DEG) in AM derived from all dietary treatment groups after 24 h of LPS stimulation. The top most significant canonical pathways in the IPA comparative analysis indicated a predictive pathway activation for IL-6 in all dietary groups (Z > 3.0), with a lower *Z* score (*Z* < 3.0) for Toll-like receptor signaling and macrophage migration inhibitory factor (MIF) regulation of immune system in the six servings of mushroom supplemented group, and IL-1 signaling for both mushroom supplemented groups (Figure 1). Eight of the top 10 DEG had a log fold reduction of at least 1.5 among pig fed mushroom compared to those on the control diet including genes encoding inflammatory cytokines (*IL6*, *IL1B*, *IL12B*), B-cell chemoattract chemokine (*CXCL13*), cytokine receptors that belong to the interleukin 1 receptor family (*ILR1*, *ILR2*), and colony stimulating factors (*CSF2*, *CSF3*), while resistin (*RETN*) and apolipoprotein A1 (*APOA1*) were upregulated relative to controls (Table 2). There was a highly significant (*p* < 0.005) LPS-induced secretion of IFNγ, IL1β, and TNFα in culture supernatants from AM isolated from pigs fed mushrooms (Table S2), but other cytokines were below the limit of detection of the assay (data not shown). The secretion of IL1β induced by LPS in cultured-AM derived from pigs fed three servings of mushroom was higher but not significantly different from controls. The level of LPS-induced TNFα from pigs fed both mushroom concentrations was also higher but not significantly different from non-stimulated AM (Figure 2A,B). The level of LPS-induced gene expression for IL1β was significantly lower in all pigs fed mushrooms compared to those on the control diet, (*p* < 0.05) (Figure 2C), while LPS-induced a non-significant lower TNFα gene expression in AM derived from pigs fed six servings of mushrooms only (Figure 2D).

### 3.3. Intestinal Response

Intestinal epithelial permeability in explanted jejunum sections was tested by *trans*-epithelial electrical resistance (TEER). Pigs from the group fed three servings of mushroom showed a non-significant increase in TEER (less permeability) when compared to those fed six servings (*p* = 0.08) with no significant difference when compared against controls (Figure S3). There were no differences in real-time PCR expression of genes associated with permeability or glucose transport in the jejunum (Table S3) or in proximal colon mucosa by RNA sequencing (data not shown).

### 3.4. Discriminant Bacterial Microbiota at Six Weeks after Diets Supplemented with Three or Six Servings of Mushrooms

Sequencing of the V1–V2 region of bacterial 16S rDNA derived from fecal samples collected at baseline and six weeks after dietary intervention were used for bacterial taxonomic analysis. No diet induced changes in alpha diversity were detected by Shannon, Chao1, or phylogenetic distance (PD) diversity indexes (Figure S4). To compare microbiome composition among dietary treatment groups, distance matrices were calculated by weighted Unifrac and visualized using principle coordinate analysis (PCoA) (Figure S5).

**Figure 3:**
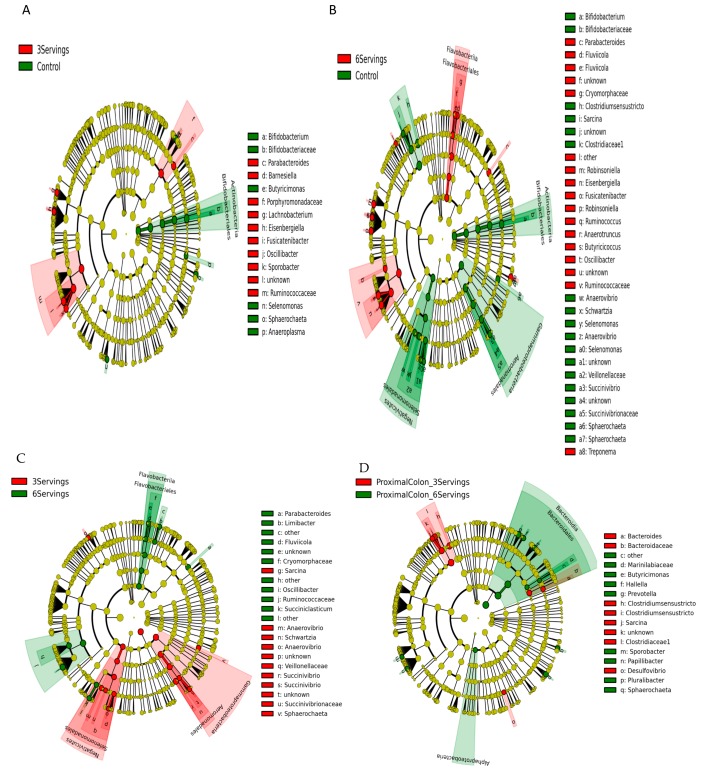
Linear discriminant analysis effect size (LEfSe) cladogram representing differentially abundant taxa in pig fecal and colonic microbiota. Enriched bacterial taxa in fecal microbiota of pigs fed three servings of white button (WB) mushrooms vs. control (**A**), six servings of WB mushrooms vs. control (**B**), three servings vs. six servings (**C**) and colonic microbiota from pigs fed three and six servings of WB mushrooms (**D**). Only taxa with linear discriminant analysis scores > 2 are presented. Each color in the pie chart represents the corresponding bacterial taxa in the legend. The LEfSe method was performed to determine individual taxa that were enriched (green) or depleted (red) within each dietary treatment comparison.

Distributions of fecal bacterial communities in fecal samples and proximal colon contents of pigs fed the control diet, or three or six servings of mushrooms in the diet clustered by time but were not affected by dietary composition at six weeks after intervention. Among 19 bacterial phyla, Firmicutes (mean 53.0%), Bacteroidetes (mean 32.0%), Spirochaetes (mean 6.0%), Proteobacteria (mean 5.0%), and Actinobacteria (mean 1.1%) were the most abundant. At the family level, *Prevotellaceae* (17.8%), *Clostridiacea* (11.0%), *Ruminococcaceae* (8.3%), *Lachnospiraceae* (6.7%) and *Spirochaetaceae* (6.5%) had the highest abundance with the genus Parabacteroides (11.0%), Streptococcus (8.8%), Prevotella (4.1%), Clostridium XI (3.1%), and Oscillobater (2.2%) at the top across all samples. Differentially abundant taxa at phylum, class, order, family, and genus levels were identified after pair-wise comparisons between dietary groups using LEfSe for biomarker (i.e., bacterial taxa) discovery applying effect size estimation. Specifically, pigs supplemented with three servings of mushrooms showed an increase in abundance of bacterial families *Ruminococcaceae* (genus Oscillibacter, Sporobacter), *Lachnospiraceae* (genus Eisenbergiella, Lachnobacterium, Fusicatenibacter) within the order Clostridiales and *Porphyromonadaceae* (genus Parabacteroides, Barnesiella) within the order Bacteroidales, and a decreased abundance in *Bifidobacteriaceae* (genus Bifidobacteria) within the phylum Actinobacteria (Figure 3A). Pigs supplemented with six servings of mushrooms, also showed an increase in *Ruminococcaceae* (Ruminococcus, Oscillibacter, Butyricicoccus), *Lachnospiraceae* (Fusicatenibacter, Robinsoniella, Eisenbergiella) within the order Clostridiales; Cryomorphaceae (genus Fluvicola) within the order Flavobacteriales, and a decreased abundance in *Clostridiaceae* (Sarcina, Clostridium sensu stricto), *Veillonellaceae* (Anaerovibrio, Schwartzia, Selenomonas), *Succinivibrionaceae* (Succinivibrio) within the phyla Proteobacteria and *Bifidobacteriaceae* within the phylum Actinobacteria (Figure 3B). Comparisons in abundance among both mushroom supplemented groups indicated an enrichment in *Ruminococcaceae* and *Cryomorphaceae* with the higher dose of mushroom supplementation (Figure 3C). Comparison of microbiota composition in the proximal colon contents of mushroom supplemented groups showed an enrichment of *Marinilabiaceae* and some members from *Prevotellaceae* (Prevotella, Hallela) within the order Bacteroidales (Figure 3D).

### 3.5. Predicted Microbial Function

**Figure 4:**
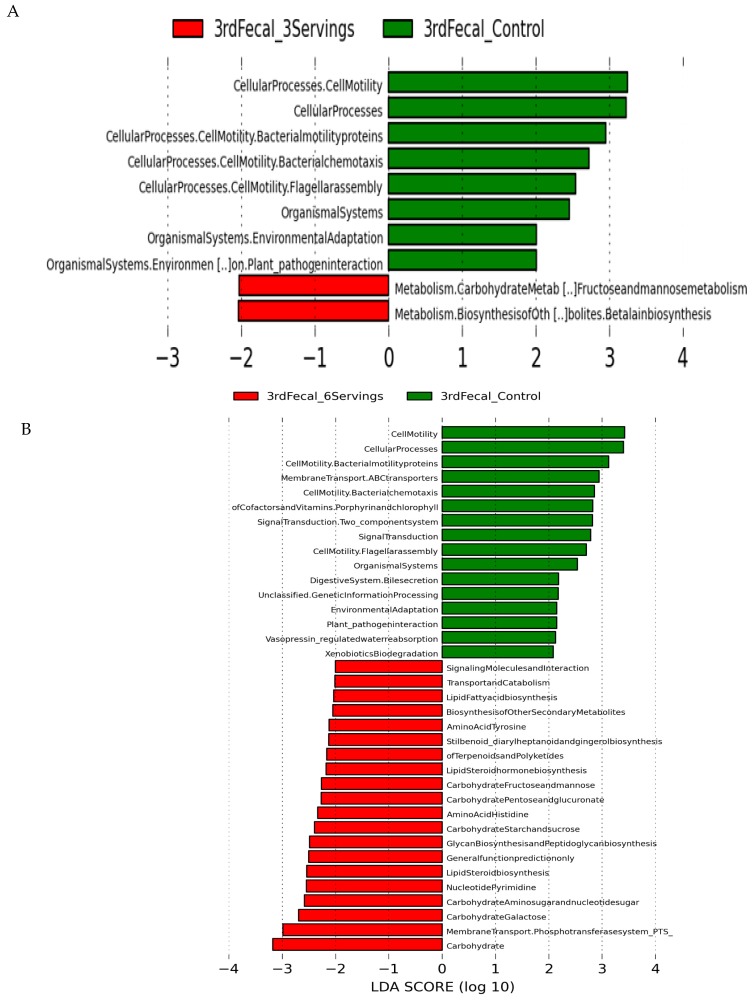

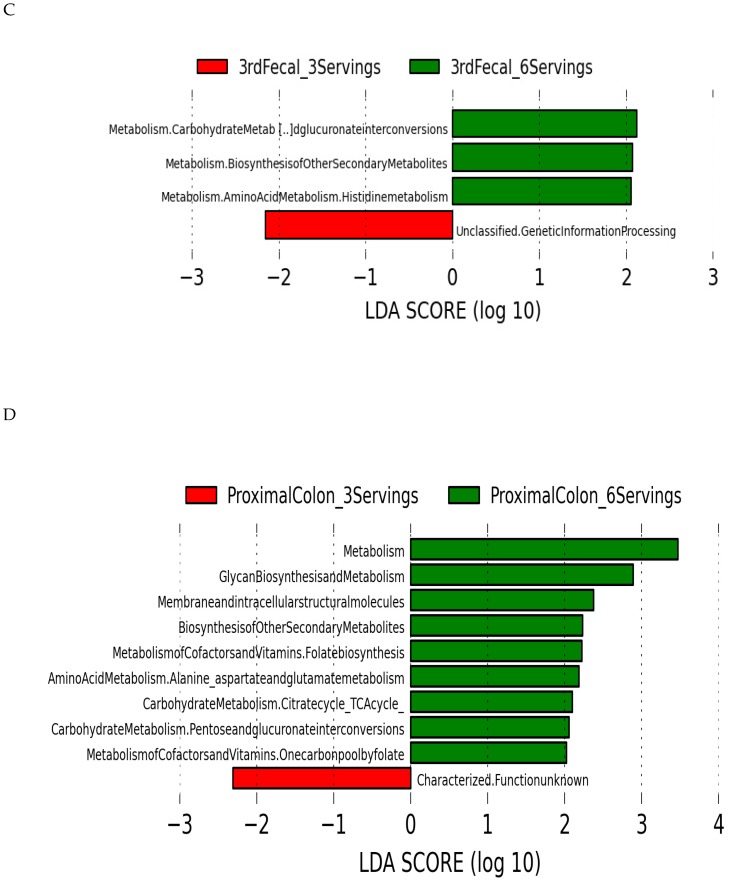
Phylogenetic Investigation of Communities by Reconstruction of Unobserved States. (PICRUSt) analysis of predicted functional pathways in the pig fecal and colonic microbiota. Imputed metabolic function results in the gut fecal microbiota of pigs fed three servings of WB mushrooms vs. control (**A**), six servings of WB mushrooms vs. control (**B**), three servings vs. six servings (**C**) and colonic microbiota from pigs fed three and six servings of WB mushrooms (**D**). Only taxa with linear discriminant analysis scores > 2 are presented. Each color in the pie chart represents the corresponding bacterial taxa in the legend.

PICRUSt was used to predict metagenomic functions from the phylogenetic profiles observed. The resulting biological pathways with an LDA score ≥ 2.0 were used to identify significantly different functions enriched in each treatment group. Carbohydrate metabolism and biosynthesis of other metabolites (e.g., betalain biosynthesis) were predicted to be increased in fecal microbiota from pigs fed three servings of mushrooms with a relative reduction in cellular processes related to bacterial chemotaxis, flagellar assembly and bacterial motility that were increased in pigs from the non-supplemented control diet group (Figure 4A). More metabolism pathways were enriched in the fecal microbiota derived from pigs fed six servings of mushrooms including pathways for carbohydrate metabolism (starch, fructose, mannose, and galactose), amino acids (histidine and tyrosine), lipids (steroid and fatty acid biosynthesis), and synthesis of secondary metabolites and glycans (Figure 4B). A comparison of predicted functional pathways in both mushroom supplemented groups indicated that pigs fed six servings of mushrooms had enriched carbohydrate metabolism including glucoronate interconversions, biosynthesis of secondary metabolites and histidine amino acid metabolism relative to pigs fed three servings of mushrooms (Figure 4C). Predicted metabolic function in the microbiota derived from the proximal colon contents indicated an enriched carbohydrate metabolism, biosynthesis of secondary metabolites, and glycan biosynthesis (Figure 4D).

## 4. Discussion

This study showed that pigs fed a six week dietary supplementation of WB mushrooms equivalent to 75 and 150 g consumed by humans positively affected the composition of the fecal and proximal colon microbiota by promoting the abundance of *Ruminococcaceae* (Oscillibacter, Butyricicoccus) and *Lachnospiraceae* (Fusicatenibacter, Robinsoniella, Eisenbergiella) families; which are known for degradation of complex plant material (cellulose and hemicellulose) in the mammalian gut [58], and are considered as beneficial given their production of butyrate. Butyrate serves as the main source of energy for colonocytes helping with the maintenance of gut homeostasis and intestinal epithelial integrity and interferes with inflammatory signals such as NF-κB [59,60]. A breakdown of epithelial integrity is found in patients with irritable bowel disease (IBD) where butyrate producing bacteria are specifically reduced when compared to healthy subjects [61]. Relative to the control group, Oscillibacter abundance was increased in pigs fed both three and six servings of WB mushrooms similar to animals fed diets rich in fermentable polysaccharides and cellulosic feeds [62,63,64]. Butyricicoccus, another producer of butyrate, has been purported to strengthen epithelial barrier function [65] and has improved good hygiene in pigs [66]. Butyricicoccus abundance was also enriched in the feces of pigs fed only the equivalent of six servings of mushrooms, suggesting a dose dependent increase in an important bacterial genus that is under-represented in the microbiota of patients with IBD compared to healthy subjects [67]. Ruminococcus abundance was also selectively increased in pigs fed six servings of mushrooms. In humans, Ruminococcus degrades plant fibers and produces acetate and succinate as major end products [68]. Non-cellulolytic ruminococcaceae have been consistently described in the gut of healthy humans with a significant reduction in abundance in patients with Crohn’s disease; suggesting a possible role in maintaining healthy gut microbiota [69]. In addition, WB mushroom supplementation enriched Fusicatenibacter, Eisenbergiella, Lachnobacterium, and Robinsoniella within the *Lachnospiraceae* family suggesting a beneficial host health effect associated with lower levels of cholesterol [70], corrections for obesity dysbiosis [71], and improved outcomes for patients with primary biliary cirrhosis [72]. Taken together, these results indicated that feeding WB mushrooms modulates the abundance and activity of specific butyrate producing bacteria from *Lachnospiraceae* and *Ruminococcaceae* in a dose dependent manner with enrichment of more substrates for carbohydrate metabolism and delivery of butyrate to the mucosa.

Relative to the non-supplemented control group, feeding pigs three and six servings of WB mushrooms reduced *Bifidobacteriacae* abundance, and enriched commensal bacteria from the families *Veillonellaceae* (Schwartzia, Selenomonas, Anerovibrio), *Succinivibrionaceae* (Succinivibrio), and *Clostridiaceae* (Sarcina, Clostridium sensu stricto) [73,74]. Pigs fed WB mushrooms had an enriched saccharolytic environment associated with bacteria from the families *Marinilabiaceae* and *Prevotellaceae* (genus Prevotella and Hallela), which are typically enriched by feeding plant rich diets with high intake of carbohydrates related to improved glucose metabolism [75].

Changes in the microbiota of pigs fed WB mushrooms are in partial agreement with a recent study of germfree and conventional mice fed lyophilized WB mushrooms [25]. These mice had increased *Prevotellaceae* in the cecum, similar to the increase in Prevotella in proximal colon of pigs fed WB mushrooms, but with additional members within the Bacteroidetes including *Porphyromonadaceae*, order Bacteroidales, Campylobacteriales within Proteobacteria, and Lactobacillales within Firmicutes with no major changes in the order Clostridiales [25]. Such differences may be explained by different dominant bacterial populations across anatomical sites (cecum vs. proximal colon vs. feces) [74], inherent features of the host, environmental factors such as diet and management or different compositions of mushrooms [1]. Moreover, the inferred microbiome metabolic data predicted an enrichment in carbohydrate metabolism, including galactose, fructose, mannose, starch, sucrose and galactose, glycan biosynthesis, tyrosine and histidine amino acid metabolism, and biosynthesis of steroids and secondary metabolites in pigs fed six servings of mushrooms.

WB mushrooms fed to pigs did not affect growth rate, carcass composition, bone mineral deposition, intestinal permeability or systemic and localized activation of mononuclear cells. However, an anti-inflammatory effect was observed in LPS-stimulated alveolar macrophages with a significant reduction in IL1β gene expression and cytokine production reflected in a lower activation of IL1-signaling in pigs fed both levels of WB mushrooms. IL1β is induced by LPS at the early stages of the immune response to bacterial infection. During inflammation, it stimulates the production of acute phase proteins in the liver and acts on the central nervous system to induce fever and prostaglandin secretion [76]. The reduced inflammatory response observed in alveolar macrophages from pigs fed WB mushrooms indicated an immune modulatory activity. However, it will need to be established if this effect is due directly to components of mushrooms or secondary metabolites derived from the intestinal microbiota. Many water, methanolic, and ethanolic extracts of mushrooms have been described to lower the production of inflammatory mediators by downregulating gene expression of these molecules [1,77]. A decrease in plasma levels of pro-inflammatory cytokines has been observed in IBD patients after oral intake of a mushroom extract isolated from a mixture of basidiomycetes mushrooms *Agaricus blazei* Murill, *Hericium erinaceum*, and *Grifola frondosa.* However, these anti-inflammatory effects have been marginal and not reproducible for all patients [17,19].

In conclusion, this study has shown that WB mushrooms act as a prebiotic that favorably affects the composition and function of the host intestinal microbiota with enrichment in carbohydrate metabolism and increased butyrate production conferring more intestinal epithelial barrier protection and reduced inflammatory stimulation.

## Figures and Tables

**Table 1:** Analysis of white button mushroom nutrient composition.

Nutrient	Per 100 g Fresh Mushroom
Protein, g/100 g	2.72
Carbohydrate, g/100 g	3.35
Fat, g/100 g	0.36
Ash, g/100 g	0.84
Moisture, g/100 g	92.72
Dietary Fiber, g/100 g	2.39
Calorie, kcal/100 g	27.53

Fresh mushroom was analyzed by Eurofin.

**Table 2:** Top differentially expressed genes in alveolar macrophages (AM) after *Salmonella typhymurium* LPS stimulation.

Gene Symbol	Gene Name	Location	Type(s)	Control_LPS		3-Serv_LPS		6-Serv_LPS		Log Difference (Control-3-Serv)	Log Difference (Control-6-Serv)	Expression Effect
				Log Ratio	FDR (*q* Value)	Log Ratio	FDR (*q* Value)	Log Ratio	FDR (*q* Value)			
*RETN*	Resistin	Extracellular space	Peptide hormone	5.117 ^a^	3.70 × 10^−5^	8.485	1.00 × 10^−4^	6.284	2.81 × 10^−5^	3.37	1.17	increased
*APOA1*	Apolipoprotein A1	Extracellular space	Lipoprotein	−5.845	3.28 × 10^−5^	−3.988	9.46 × 10^−5^	−4.057	4.70 × 10^−5^	1.86	1.79	increased
*CSF3*	Colony stimulating factor 3	Cytoplasm	Growth factor	8.816	3.20 × 10^−6^	7.058	2.00 × 10^−5^	6.982	4.97 × 10^−6^	−1.758	−1.834	reduced
*IL12B*	Interleukin 12B	Plasma membrane	Cytokine	8.486	6.51 × 10^−4^	5.527	2.57 × 10^−3^	5.944	2.68 × 10^−4^	−2.959	−2.542	reduced
*IL6*	Interleukin 6	Extracellular space	Cytokine	8.428	5.35 × 10^−5^	5.587	3.45 × 10^−4^	5.05	1.30 × 10^−4^	−2.841	−3.378	reduced
*IL1B*	Interleukin 1 beta	Plasma membrane	Cytokine	8.289	1.58 × 10^−5^	7.192	1.17 × 10^−4^	6.985	3.65 × 10^−5^	−1.097	−1.304	reduced
*CSF2*	Colony stimulating factor 2	Plasma membrane	Growth factor	7.537	4.60 × 10^−5^	5.661	2.04 × 10^−4^	5.344	4.28 × 10^−5^	−1.876	−2.193	reduced
*CXCL13*	C-X-C motif chemokine ligand 13	Cytoplasm	Chemokine	4.92	4.31 × 10^−4^	3.869	6.78 × 10^−3^	3.51	5.49 × 10^−3^	−1.051	−1.41	reduced
*IL1R1*	Interleukin 1 receptor type 1	Plasma membrane	IL1 type receptor	3.623	3.55 × 10^−4^	1.798	2.26 × 10^−02^	1.61	1.86 × 10^−02^	−1.825	−2.013	reduced
*IL1R2*	Interleukin 1 receptor type 2	Cytoplasm	IL1 type receptor	3.532	9.77 × 10^−5^	1.62	1.83 × 10^−02^	2.226	1.04 × 10^−3^	−1.912	−1.306	reduced

^a^ Log fold change of genes: *RETN* (Resistin), *APOA1* (Apolipoprotein A1), *CSF3* (Colony stimulating factor), *IL12B* (Interleukin 12B), *IL6* (Interleukin 6), *IL1B* (Interleukin 1 beta), CSF2 (Colony stimulating factor 2), CXCL13 (Chemokine C-X-C motif ligand 13), IL1R1 (Interleukin1 receptor type 1), IL1R2 (Interleukin1 receptor type 2) after Lipopolysaccharide (LPS) stimulation of alveolar macrophages (AM) collected from pigs fed a control or mushroom supplemented diets (3_serv or 6_servings) relative to matching AM control group with no stimulation with an adjusted false discovery rate (FDR) < 0.05.

## Supplementary Materials

The following are available online at http://www.mdpi.com/2072-6643/10/11/1721/s1, Figure S1: Body weight change, body composition by DXA and bone mineral content, Figure S2: Immune staining of ileum Peyer’s patch cells and alveolar macrophage phagocytosis, Figure S3: Effect of dietary treatment on intestinal epithelial permeability, Figure S4: Bacterial community alpha diversity, Figure S5: Bacterial community beta diversity, Table S1: Diet composition, Table S2: Alveolar macrophage cytokine production, Table S3: Jejunum gene expression analysis by RT-PCR.

## Abbreviations

QIIME	Quantitative Insights into Microbial Ecology
LDA	Linear Discriminant Analysis
LEfSe	Effect Size
PICRUSt	Phylogenetic Investigation of Communities by Reconstruction of Unobserved States
