# Supplementary material for: The Effect of Dietary Mushroom Agaricus bisporus on Intestinal Microbiota Composition and Host Immunological Function
Source: Nutrients. 2018 Nov 9;10(11):1721. doi: 10.3390/nu10111721 (PMC6266512; doi:10.3390/nu10111721)
Supplement: Supplementary file 1 [file nutrients-10-01721-s001.zip › F_Figure S1_Body weight change body composition andbone mineral content.pptx]

## Slide 1
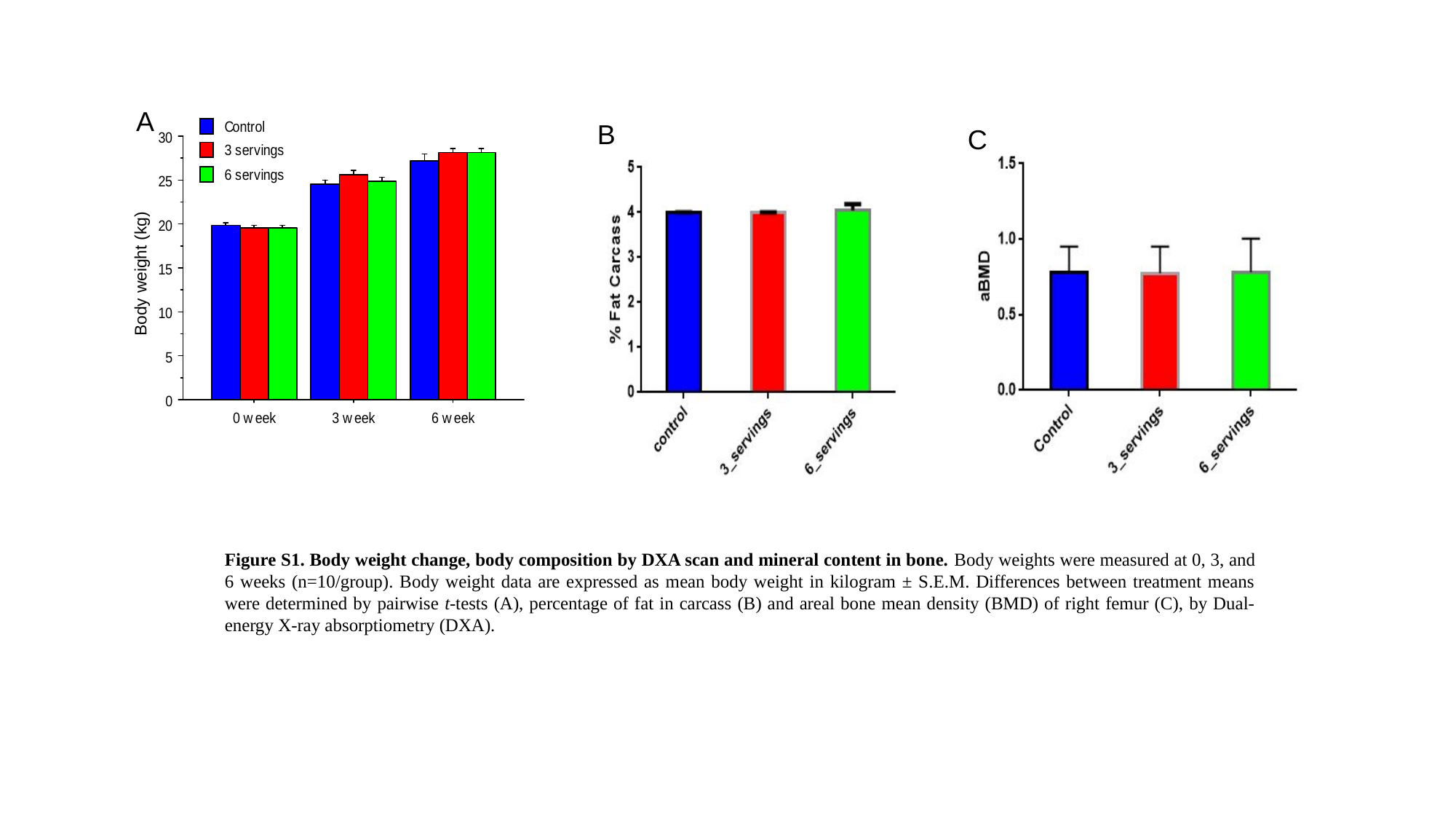

A
Body weight (kg)
B
C
Figure S1. Body weight change, body composition by DXA scan and mineral content in bone. Body weights were measured at 0, 3, and 6 weeks (n=10/group). Body weight data are expressed as mean body weight in kilogram ± S.E.M. Differences between treatment means were determined by pairwise t-tests (A), percentage of fat in carcass (B) and areal bone mean density (BMD) of right femur (C), by Dual-energy X-ray absorptiometry (DXA).
