# Supplementary material for: The Effect of Dietary Mushroom Agaricus bisporus on Intestinal Microbiota Composition and Host Immunological Function
Source: Nutrients. 2018 Nov 9;10(11):1721. doi: 10.3390/nu10111721 (PMC6266512; doi:10.3390/nu10111721)
Supplement: Supplementary file 1 [file nutrients-10-01721-s001.zip › F_Figure S2_Immunostaining of isolatedIleal Peyer pathches and macrophage function.pptx]

## Slide 1
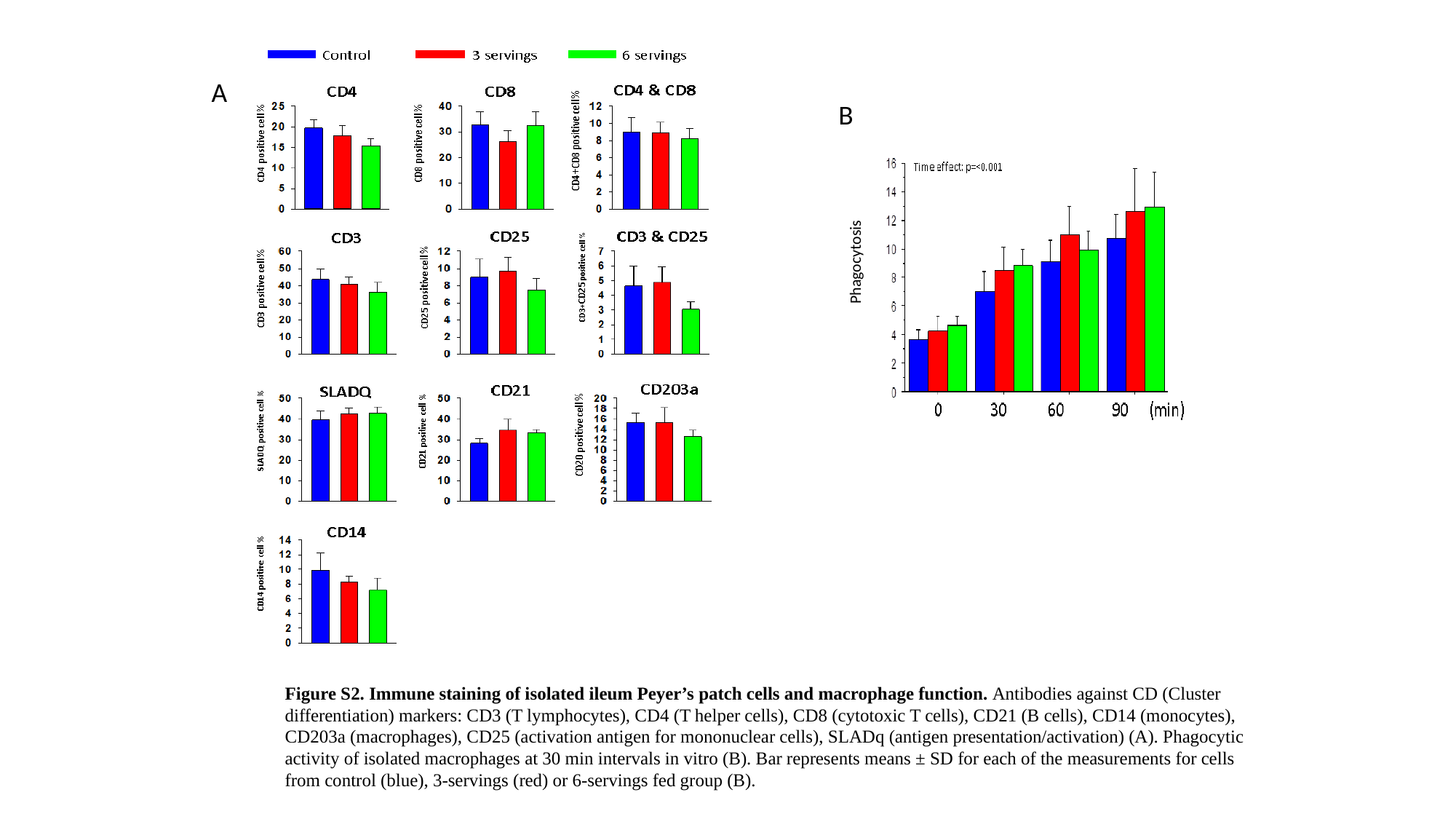

A
B
Phagocytosis
Figure S2. Immune staining of isolated ileum Peyer’s patch cells and macrophage function. Antibodies against CD (Cluster differentiation) markers: CD3 (T lymphocytes), CD4 (T helper cells), CD8 (cytotoxic T cells), CD21 (B cells), CD14 (monocytes), CD203a (macrophages), CD25 (activation antigen for mononuclear cells), SLADq (antigen presentation/activation) (A). Phagocytic activity of isolated macrophages at 30 min intervals in vitro (B). Bar represents means ± SD for each of the measurements for cells from control (blue), 3-servings (red) or 6-servings fed group (B).
