# Supplementary material for: The Effect of Dietary Mushroom Agaricus bisporus on Intestinal Microbiota Composition and Host Immunological Function
Source: Nutrients. 2018 Nov 9;10(11):1721. doi: 10.3390/nu10111721 (PMC6266512; doi:10.3390/nu10111721)
Supplement: Supplementary file 1 [file nutrients-10-01721-s001.zip › F_Figure S3_ Effect of dietary treatment on intestinal epithelial permeability.pptx]

## Slide 1
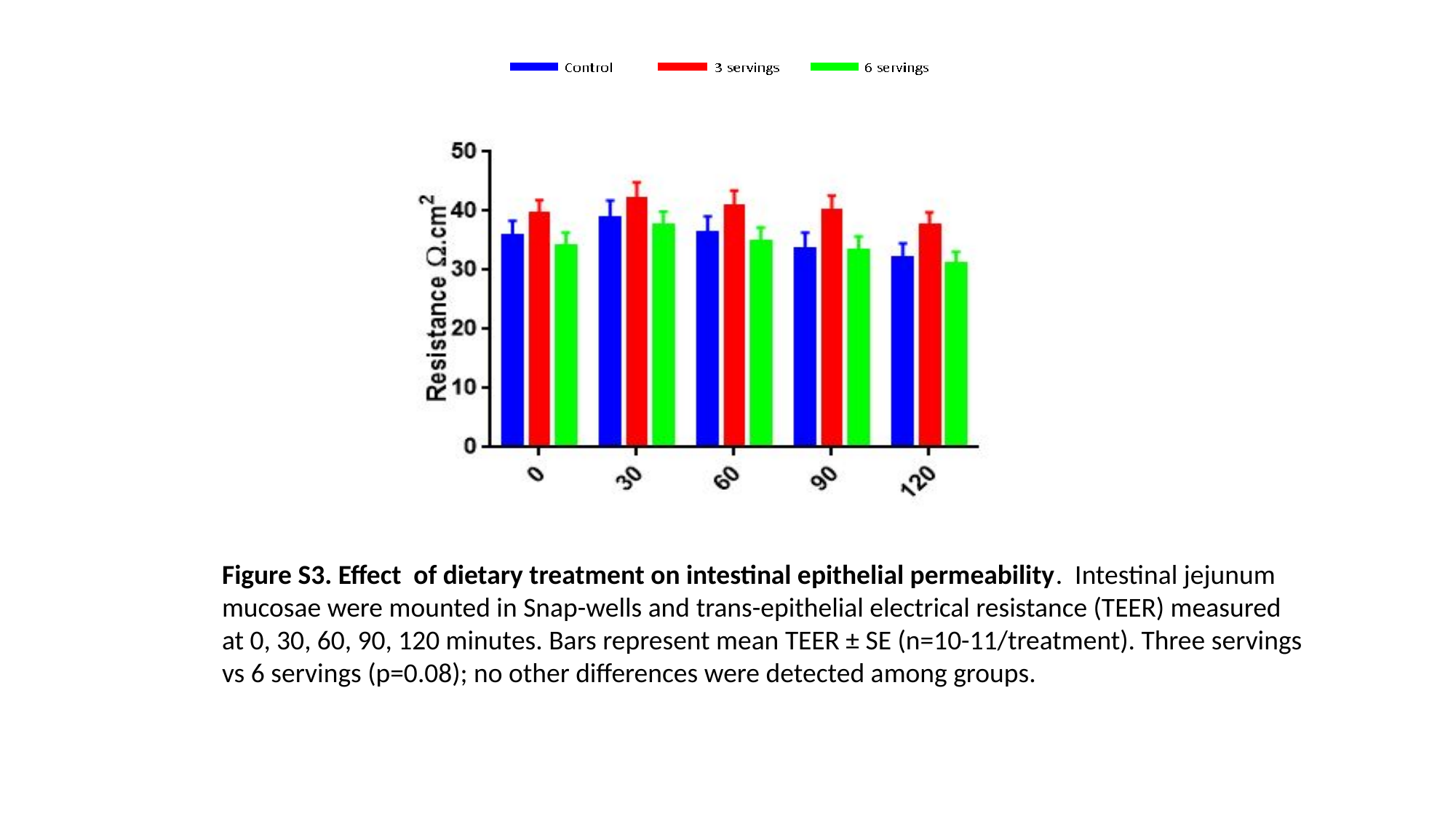

Figure S3. Effect of dietary treatment on intestinal epithelial permeability. Intestinal jejunum
mucosae were mounted in Snap-wells and trans-epithelial electrical resistance (TEER) measured
at 0, 30, 60, 90, 120 minutes. Bars represent mean TEER ± SE (n=10-11/treatment). Three servings
vs 6 servings (p=0.08); no other differences were detected among groups.
