# Supplementary material for: The Effect of Dietary Mushroom Agaricus bisporus on Intestinal Microbiota Composition and Host Immunological Function
Source: Nutrients. 2018 Nov 9;10(11):1721. doi: 10.3390/nu10111721 (PMC6266512; doi:10.3390/nu10111721)
Supplement: Supplementary file 1 [file nutrients-10-01721-s001.zip › F_Figure S4_Bacterial community alpha diversity.pptx]

## Slide 1
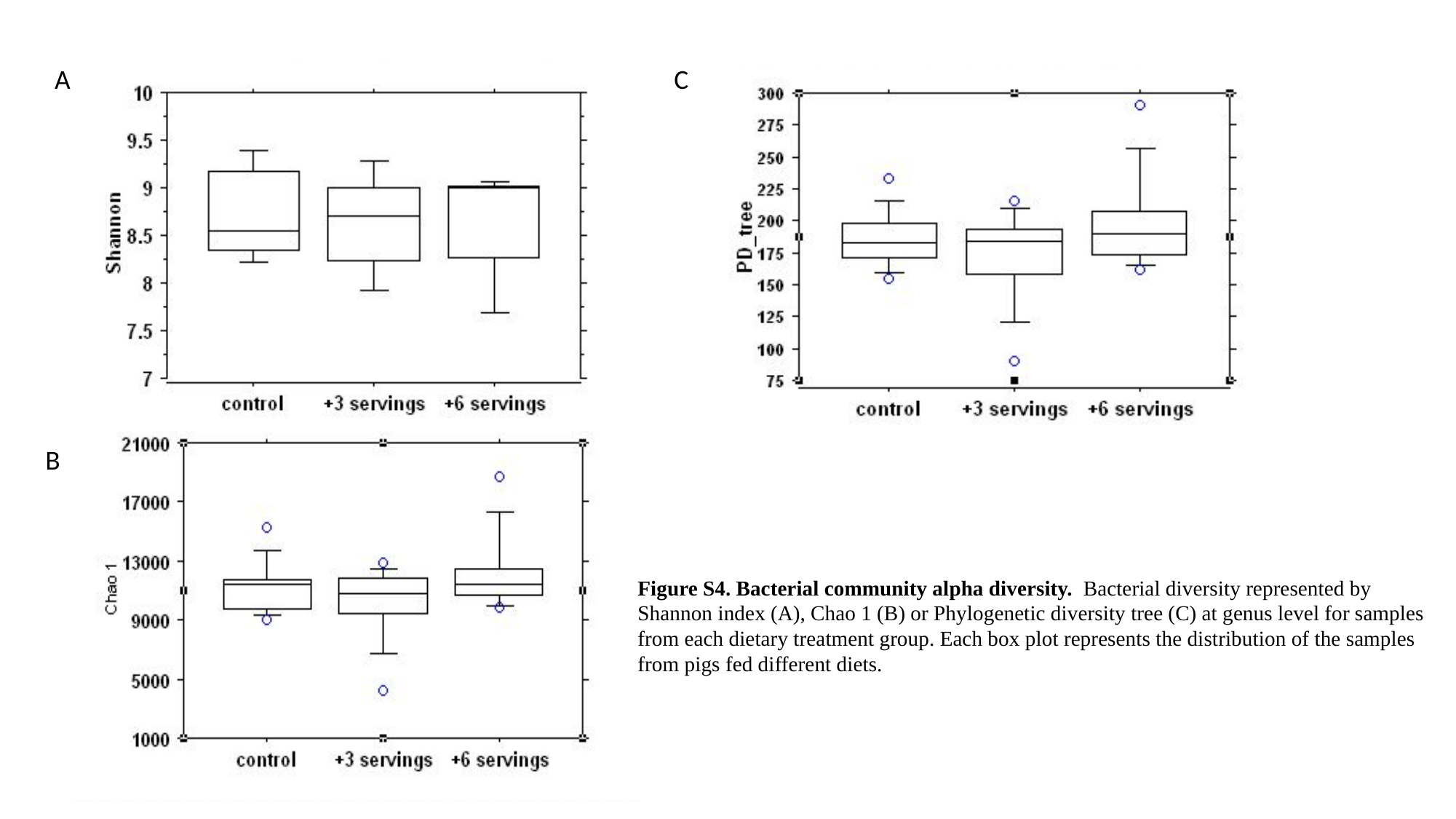

A
C
B
Figure S4. Bacterial community alpha diversity. Bacterial diversity represented by
Shannon index (A), Chao 1 (B) or Phylogenetic diversity tree (C) at genus level for samples
from each dietary treatment group. Each box plot represents the distribution of the samples
from pigs fed different diets.
