# Supplementary material for: The Effect of Dietary Mushroom Agaricus bisporus on Intestinal Microbiota Composition and Host Immunological Function
Source: Nutrients. 2018 Nov 9;10(11):1721. doi: 10.3390/nu10111721 (PMC6266512; doi:10.3390/nu10111721)
Supplement: Supplementary file 1 [file nutrients-10-01721-s001.zip › F_Figure S5_ Bacterial community beta diversity.pptx]

## Slide 1
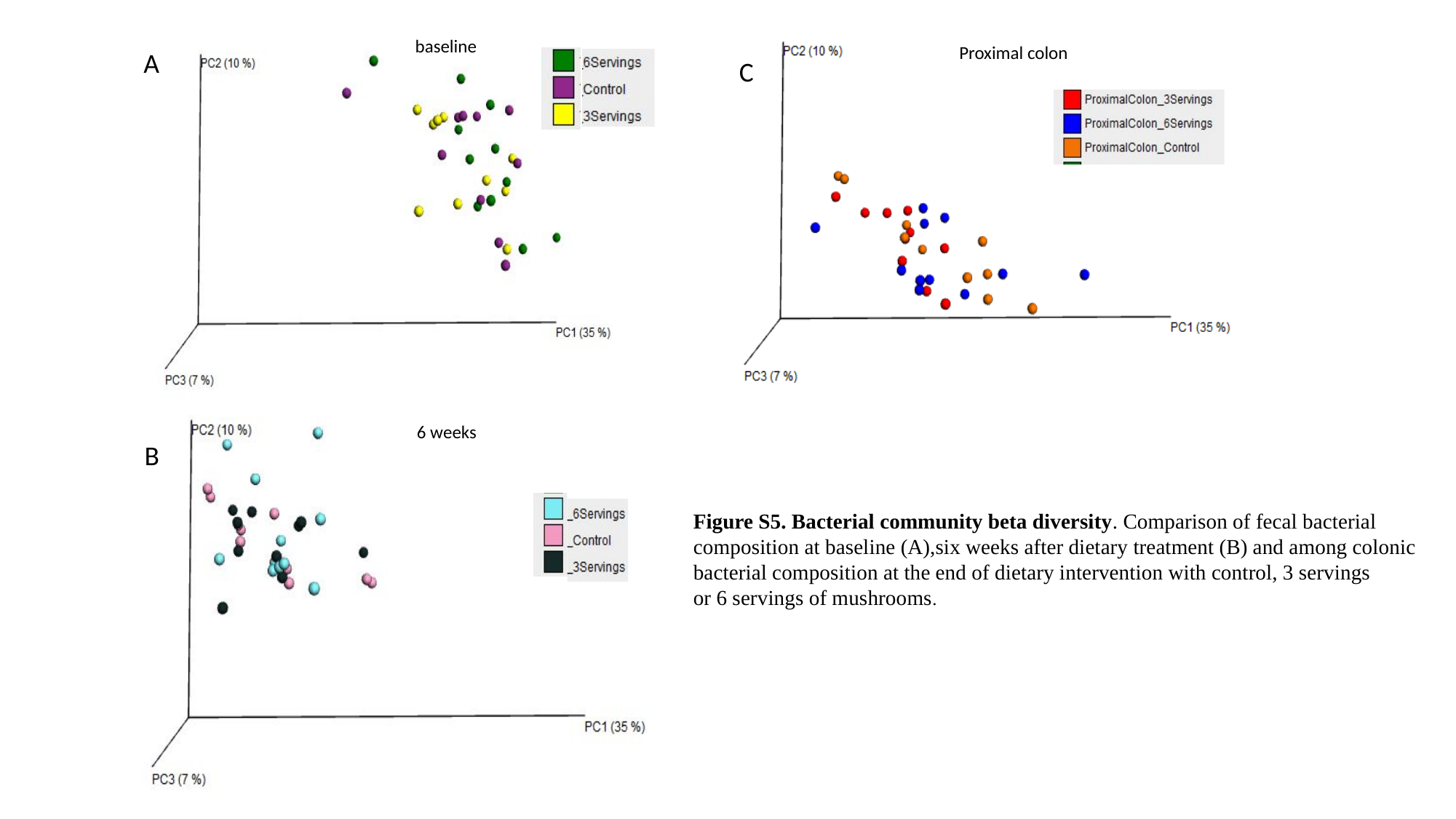

baseline
Proximal colon
A
C
6 weeks
B
Figure S5. Bacterial community beta diversity. Comparison of fecal bacterial
composition at baseline (A),six weeks after dietary treatment (B) and among colonic
bacterial composition at the end of dietary intervention with control, 3 servings
or 6 servings of mushrooms.
